# History of Newcastle disease in South Africa

**DOI:** 10.4102/ojvr.v84i1.1306

**Published:** 2017-02-24

**Authors:** Celia Abolnik

**Affiliations:** 1 Department of Production Animal Studies, University of Pretoria, South Africa

## Abstract

Poultry production in South Africa, a so-called developing country, may be seen as a gradient between two extremes with highly integrated commercial enterprises with world-class facilities on one hand and unimproved rural chickens kept by households and subsistence farmers on the other. Although vaccination against Newcastle disease is widely applied to control this devastating infection, epizootics continue to occur. Since the first official diagnosis in 1945, through the sporadic outbreaks of the 1950s and early 1960s, to serious epizootics caused by genotype VIII (late 1960s–2000), genotype VIIb (1993–1999), genotype VIId (2003–2012) and most recently genotype VIIh (2013 to present), South Africa’s encounters with exotic Newcastle disease follow global trends. Importation – probably illegal – of infected poultry, poultry products or exotic birds and illegal swill dumping are likely routes of entry. Once the commercial sector is affected, the disease spreads rapidly within the region via transportation routes. Each outbreak genotype persisted for about a decade and displaced its predecessor.

## Introduction

Newcastle disease (ND) is a highly contagious and lethal avian disease caused by certain strains of avian paramyxovirus type 1 (aPMV-1) that is transmitted by the faecal–oral route in avian species. aPMV-1 is a member of the Avulavirus genus, viruses that have a membrane-encapsulated single-stranded RNA genome of negative polarity (Alexander 1997; Mayo 2002). aPMV-1 is split into two classes: Class I consists of aPMV-1 viruses commonly isolated from wild birds, whereas the stains infecting poultry fall within Class II (Diel et al. 2012). Each class is further divided into genotypes; the current classification is summarised in Table 1 along with pertinent characteristics of each genotype. These genotypes have emerged over the course of several decades, due in part to the inherent propensity of RNA viruses to evolve rapidly, as well as immune pressure that drives strain selection within the host (Miller et al. 2009).

**TABLE 1:** Current classification of avian paramyxovirus type 1 (Newcastle disease virus).

Class	Genotype	Sub-genotype	Characteristics
Class I	I	1a	Lentogenic viruses Primarily aquatic birds, but also poultry
		1b	
		1c	
		U	
Class II	I	Ia	Lentogenic viruses E.g. Queensland/ V4, I-2 and Ulster 2C/67 strains. Some strains used as vaccines
		Ib	
		Ic	
	II	-	Mixed pathotypes E.g. Hitchner B1/47, LaSota/46, VG/GA, F-strain, Komarov and Roakin strains First panzootic (1920s–1950s), originated in South East Asia. Some strains used as vaccines
	III	-	Velogenic viruses E.g. Miyadera/51 and Mukteswar strains First panzootic (1920s–1950s), originated in South East Asia
	IV	-	Velogenic viruses E.g. Herts/33, Italien/44 and Texas GB/48 strains First panzootic (1920s–1950s), originated in South East Asia
	V	Va	Velogenic viruses Second panzootic (Emerged 1960s–1970s), circulated in England, Europe and California Frequently isolated in South and Central American poultry, cormorants in North America
		Vb	
		Vc	
		Vd	
	VI	VIa	Mixed pathotypes Third panzootic - Pigeons Fatal in pigeons and doves, intermediate virulence in poultry Originated in the Middle East in 1980s Global distribution due to racing pigeon sport
		VIb	
		VIc	
		VIe	
		VIf	
		VIg	
		VIh	
		VIi	
	VII	VIIb	Velogenic viruses Fourth panzootic Originated in Taiwan and Indonesia in the 1980s Pathogenic to multiple avian species including waterfowl Currently, the predominant global genotype, outbreaks in European Union, Middle East, Asia, southern Africa and South America
		VIId	
		VIIe	
		VIIf	
		VIIg	
		VIIh	
		VIIi	
	VIII	-	Velogenic viruses Circulated in South Africa, Argentina and East Asia, 1960s–2000
	IX	-	Velogenic viruses Early genotype, emerged between 1930s and 1960s in East Asia, sporadic Asian outbreaks
	X	-	Lentogenic viruses Isolated from waterfowl and shorebirds, North America, late 1980s to present
	XI	-	Velogenic viruses Originated in and restricted to Madagascar, possibly derived from genotype IV
	XII	-	Velogenic viruses Circulated in East Asia and South America, China (geese)
	XIII	XIIIa	Velogenic viruses Circulated in Pakistan, Iran, Russia, India, Sweden and Burundi
		XIIIb	
	XIV	XIVa	Velogenic viruses Originated in and restricted to West and Central Africa Outbreaks in poultry with spill-over to wild birds
		XIVb	
	XV	-	Velogenic viruses Outbreaks in Chinese chickens and geese
	XVI	-	Velogenic viruses Outbreaks in Central and South American poultry
	XVII	XVII	Velogenic viruses Originated in and restricted to West and Central Africa Outbreaks in poultry with spill-over to wild birds
		XVIIb	
	XVIII	XVIIa	Velogenic viruses Originated in and restricted to West and Central Africa Outbreaks in poultry with spill-over to wild birds
		XVIIb	

*Source:* Compiled from Rui et al. (2010); Maminiaina et al. (2010), Diel et al. (2012); De Almeida et al. (2013); Miller et al. (2015) and Dimitrov et al. (2016)

Genotypes are defined by > 10% mean nucleotide differences of the fusion protein gene with a bootstrap value at the defining node of > 60%. Sub-genotypes are defined by a mean genetic distance of 3%–10% plus a bootstrap value of > 60% at the defining node.

Many avian species have been diagnosed with aPMV-1 infection, but clinical signs tend to vary with the pathogenicity of the isolate and the species of the bird. Chickens are highly susceptible to aPMV-1 infection; strains are therefore further classified based on the symptoms they cause in this species. Lentogenic strains typically cause subclinical infections or mild respiratory disease, with coughing, gasping, sneezing and rales. Mesogenic strains potentially cause acute respiratory disease and neurologic signs in some chickens, but the mortality rate is usually low. Velogenic strains cause severe, often fatal disease. Most poultry infected with velogenic aPMV-1 (synonymous with ‘ND’) appear lethargic and inappetant with conjunctivitis and ruffled feathers. Additional symptoms often include watery, greenish or white diarrhoea, respiratory signs, cyanosis and swollen heads. Neurologic signs include tremors, clonic spasms, paresis or paralysis of the wings and/or legs, torticollis and circling. Egg production often declines dramatically, and eggs may be misshapen, abnormally coloured and rough or thin-shelled, with watery albumin. Sudden death, with no or few symptoms, is also common with velogenic aPMV-1 strains (Alexander 1997). The intracerebral pathogenicity index (ICPI) provides a relative measure of a virus’ pathogenicity. The most virulent viruses will give indices that approach the maximum score of 2.0, whereas lentogenic and asymptomatic enteric strains will give values close to 0.0 (OIE 2012). The presence of multiple basic amino acids at the fusion (F) protein cleavage site (F_0_) is another important indicator of viral virulence (Alexander 1997).

ND is a severe global problem affecting poultry production. In Africa its effect is particularly severe on backyard poultry flocks. Poultry production in South Africa, a so-called developing country, may be seen as a gradient between two extremes, with highly integrated commercial enterprises with world-class facilities on one hand and unimproved rural chickens kept by households and subsistence farmers on the other. Although vaccination is widely applied to control the disease, epizootics continue to occur. A description of South Africa’s experience with this devastating disease in the context of global outbreaks follows.

### Early global and South African outbreaks

ND may have been prevalent in Scotland as early as 1898, when a disease decimated domestic fowl in the Scottish northwest region (Maminiaina et al. 2010). The first confirmed outbreaks of ND occurred in 1926 in Java, Indonesia, and in 1927, in Newcastle-upon-Tyne, England, but the disease could also have been present in Korea in 1924 (Konno, Ochi & Hashimoto 1929). The following letter in 1892 to Agricultural Journal (South Africa) may, however, be the earliest account yet:

Can any of your readers give me some information as to the causes, prevention, and treatment of a disease that from time to time attacks my fowls, and seems to resist all known treatment. The disease apparently begins with a cough, and gasping breathing, the fowl trembles all over, and when it sits down one leg is stretched in front the other behind the body. There seems to be a gradual paralysis of both legs. The patient has all along an excellent appetite, which is maintained to the very end of the disease-invariably death. Some of the cases make gurgling noise when breathing, and they generally live in this dying state for months. Aloes, rusty water, lime, and paraffin-remedies recommended by “fowl” authorities have all been tried-as well as homoeopathic doses of aconite, without benefit. I have two English imported canaries ill, and the symptoms are very similar. As the canaries are valuable birds I am anxious to know of a cure. (Mr T. Duncan Greenlees, Fort England, Grahamstown, Query No. 221- Fowl-sickness 1892)

Another example of possible ND citing extracts from a letter to the Transvaal Agricultural Journal in 1903:

I have a considerable number of mixed breed fowls running on my place here. They suffer from various diseases known to us locally as ‘Lame-sickness’, ‘Eye-sickness’, ‘Sore Throat,’ ‘Sore Nostrils,’ &c., the right names of which we do not know. ‘Lame-sickness’, the fowl becomes unable to walk, and appears to have paralysis of the legs, the wings droop, the foeces [*sic*] are loose, weakness sets in and the bird dies … Sore Throat,’ ‘Sore Nostrils,’ &c.-The fowl eventually chokes, suffocates and dies. It resembles a child’s croup’ … There is another disease, which is difficult to describe. The fowl appears to get into a fit, dances about, and suddenly falls down and dies. (Ada Rosenbloom, Hamans Kraal 1903)

Although many of the symptoms described in these two cases are not pathognomonic for ND, and indeed, the description of unilateral paresis or paralysis is suggestive of a Marek’s disease infection, the presence of neurological signs makes ND a strong possibility. ND may therefore have been present in South Africa since at least the late 1800s.

### Newcastle disease outbreaks in South Africa, 1945–1970s

ND was officially diagnosed for the first time in South Africa in 1945, after a severe disease with respiratory, nervous and intestinal symptoms, and high mortalities swept through poultry in the Natal (KwaZulu-Natal) province. The diagnosis was made by serum neutralisation tests performed at Weybridge in England. Kashscula believed that ND was already present in the port of Durban in September 1944 and because the symptoms and post-mortem findings in Natal so closely resembled those described by Hudson in Mombassa, Kenya in 1935, it was ‘almost certain’ that the disease had been brought by ship from some harbour on the East Coast of Africa. Hudson considered that the infection had spread south to Lindi and there were persistent rumours suggesting that the entire African East Coast had been affected (Kaschula et al. 1945). In retrospect, this outbreak appears to have been part of a panzootic that swept through Italy, Palestine and the whole of central Europe during the Second World War (Kluge 1964). The 1944/1945 outbreak in South Africa was confined almost entirely to the sugarcane belt of Natal. The free-ranging flocks of the Indian population suffered the heaviest losses. The disease was eventually stamped out, but 100 000 fowl are estimated to have perished. It was strongly suspected that the Mombassa outbreak in 1935 was not the first African case of ND (Kaschula et al. 1945).

Confirmed ND appeared in Cape Town’s Windermere Township in July, 1949. By September it had spread to Port Elizabeth and De Aar in the Cape province. By January 1950 ND had spread to Natal and was also confirmed in Johannesburg. More outbreaks flared up in Durban during January of 1951 and 1953. With the application of strict control measures, the disease was suppressed until early October, 1954 when ND reappeared in Johannesburg. By the end of December 1954 the disease had been stamped out, but it was estimated that more than 300 000 fowls perished in these outbreaks of the early 1950s. Vaccines had recently been developed and more than a million birds were vaccinated with an attenuated live virus (De Kock 1954).

In July 1960, the mild lentogenic type of ND, termed the ‘American type’, was diagnosed in South Africa for the first time. The strain of virus was so mild that symptoms of infection were apparent only to the careful observer (Kluge 1964). Velogenic ND re-emerged in the northern outskirts of Pretoria in August 1961 and reached Evaton in November, where it re-emerged in June 1962. Thereafter, an intensive control campaign was successfully concluded and no further outbreaks occurred for several years (Kluge 1964).

In 1967, a serious ND outbreak occurred on a farm in the Cape Flats near Cape Town, despite control measures that included quarantine and vaccination. By July 1968, the Bellville and Stellenbosch regions were also affected, and the disease spread throughout the district. This strain was referred to as the ‘Asiatic type’, whereas the milder ‘American type’ was simultaneously circulating in the Transvaal (Gauteng), Natal and Eastern Cape provinces (Oosthuizen 1979). The outbreaks caused serious losses while they lasted, but the control measures instituted by the State (quarantine and other zoo-sanitary measures, in conjunction with immunisation of all birds in the surrounding and proclaimed areas) seemed to effectively control the disease and eliminated the infection in each instance. The live mesogenic Komarov strain was used as a vaccine and no immunisation against ND was practised or allowed at any time except during outbreaks of the disease and then only under State supervision. It was believed that the disease was not enzootic in South Africa and the localised nature of the outbreaks indicated that the infections were probably introduced from some unknown external sources. Therefore, ND was not considered to be of major importance for South Africa (De Almeida et al. 2013; Kluge 1964).

South Africa experienced one of its most severe ND epidemics from 1970 to 1972. The rapid expansion of the industry since 1960 had created large concentrations of susceptible poultry. Farms accommodating hundreds of thousands of chickens were established in close proximity to one another and near the urban markets. The method of eradication previously employed to control ND had become impractical because of the rapid dissemination of the infection in certain regions (Coetzee 1980). The first outbreak occurred in the town of Potgietersrus (Mokopane), and later, in September 1970 on the outskirts of Pretoria in backyard poultry. By December, one of the major commercial broiler operations in Johannesburg was affected. The failure of the campaign to eradicate ND was attributed to two reasons. Firstly, there was a lack of adequate funds for slaughtering out with compensation – as had previously been applied – and secondly, as a result of the development of huge broiler operations. The industry suffered great economic losses and by 1971 ND was believed to be enzootic in the country. By 1974 outbreaks of ND had subsided and occurred only in unvaccinated birds on small poultry farms and among unvaccinated poultry kept in backyards. Outbreaks were reported fairly frequently in consignments of imported birds at the quarantine station at the international airport in Johannesburg. These consignments were evidently infected while they were handled by dealers prior to shipment, since all birds, virtually without exception, developed ND from 3 to 7 days after their arrival in South Africa. Vaccination against ND became a common practise at the airport quarantine station (Anonymous, SAPV-Pluimveebulletin, February 1974). Subsequent to the severe outbreak of the 1970s, only small sporadic outbreaks occurred over the next two decades.

### Genetic characterisation of Newcastle disease strains in South Africa begins

The sources of sporadic ND outbreaks since the 1950s were never resolved and it was presumed that the periodic outbreaks were spill-overs from an unknown reservoir, which some suggested to be village chickens, but wild birds were also suspected (Verwoerd 1995b). The national veterinary laboratories at Allerton in KwaZulu-Natal, Stellenbosch in the Western Cape and the University of Pretoria routinely isolated aPMV-1s from the national flock during outbreaks. To resolve the molecular epidemiology of ND in South Africa, a project was started at the Onderstepoort Veterinary Institute in Pretoria, whereby all the available aPMV-1 isolates were subjected to reverse-transcription polymerase chain reaction (PCR) amplification of a portion of the F gene, followed by DNA sequencing and phylogenetic analysis (Abolnik 2007). In the course of these molecular investigations, virulent genotypes were identified (described below) as well as genotype I and II vaccine derivatives. Genotype VI was, and still is, occasionally isolated from pigeons (and in some cases, chickens), but will not be discussed in this review. Thus the strains responsible for causing sporadic epidemics in South African poultry since the 1960s were identified as follows.

### Genotype VIII outbreak (1960s–1994; 2000)

Genotype VIII is one of the smallest exotic ND groups in the world and is likely to have originated in Southeast Asia. These strains are velogenic and were isolated in poultry in Singapore and western China between 1979 and 1985, Argentina in 1970, Japan in 1991, Taiwan in 1995 and Italy in 1994 (Aldous et al. 2003; Liang et al. 2002; Mase et al. 2002; Rui et al. 2010; Tsai et al. 2004). In South Africa, genotype VIII was isolated in 1968, 1974 and in the early 1990s up to 1994. Herczeg and co-workers suggested that this genotype was enzootic in South Africa since the 1960s (Herczeg et al. 1999). Most isolations of genotype VIII were made in the 1990s in the KwaZulu-Natal province, one of the largest and most intensive poultry-producing areas in South Africa, but two more genotype VIII strains were isolated in the latter part of the decade. These late isolates did not appear to be as virulent as those prior to 1994 since the only clinical signs reported were a slight drop in egg production and nasal excretions. Whether this was due to improved and widespread vaccination in the 1990s or attenuation is unknown. The reservoir of genotype VIII in South Africa between 1994 and 2000 is also unknown since despite intensive active surveillance and the ongoing molecular epidemiological study, no genotype VIII viruses were isolated by the national laboratories during this period. No genotype VIII strains have been detected in South Africa since June 2000 (Abolnik 2007; unpublished laboratory data).

### Genotype VIIb (1993–1999)

In June 1993, a neuro/respirotropic NDV was isolated from an outbreak at a large commercial poultry producer near Hartebeespoort, Gauteng province. Within 6 months ND had spread throughout southern Africa causing devastating losses in all types of poultry. An estimated one million mortalities per week were recorded at the peak of the outbreak. The major outbreak of 1993/1994 was eventually brought under control by improved vaccination procedures and biosecurity measures, but sporadic outbreaks continued to occur for the remainder of the decade (Huchzermeyer & Gerdes 1993; Verwoerd 1995a, 1995b). In retrospect, the incurrence of genotype VIIb into South Africa in the early 1990s was concurrent with the rise of this pandemic strain in many parts of East Asia and Europe in the 1990s. Extensive phylogenetic analysis demonstrated that the genotype VIIb strain responsible for the outbreaks in South Africa originated in Southern Europe (Abolnik 2007) and not the opposite, as was originally reported (Herczeg et al. 1999). Genotype VIIb may even have been introduced from abroad into South Africa on multiple occasions since a genotype VIIb virus had been isolated from an ostrich in South Africa in 1991, 2 years prior to the outbreak index case at Hartebeespoort, yet the ostrich virus shared only 97% nucleotide sequence identities with the early South African genotype VIIb. The South African genotype VIIb outbreak strains from 1993, however, shared 100% sequence identity with Spanish (1990) and Portuguese (1991) isolates (Abolnik 2007). It is possible that day-old chicks, eggs or unprocessed poultry products were imported into South Africa from Europe, or via a neighbouring country trading with Europe, before the infection was reported there. Genotype VIIb was also present in Mozambique in 1993 (Herczeg et al. 1999) and reached Botswana. Genotype VIIb was not detected in South Africa after 1999 (Abolnik 2007).

### Genotype VIId (2003–2012)

Generally, aPMV-1 strains infect waterfowl such as geese and ducks without causing overt clinical symptoms. These species therefore usually act only as carriers of the virus. However, ND outbreaks have occurred frequently in Chinese geese since 1997 (Alexander 1997; Takakuwa et al. 1998; Yin & Liu 1997). In 1999, an outbreak of ND in Shanghai goose flocks caused moderate morality rates of 10% – 20% in adult geese but up to 100% mortalities were observed in geese under 2 weeks of age. A novel virus isolate, SF02, was determined to be the etiological agent of these outbreaks. It was named ‘Goose paramyxovirus’ and was subsequently classified as genotype VIId. Genotype VIId is highly pathogenic to chickens, pigeons, partridges and ducks. ICPIs of 1.80–1.94 have been recorded, but chickens inoculated with either live or inactivated LaSota vaccines were fully protected against challenge with genotype VIId (Jinding et al. 2005; Liu et al. 2003; Zou et al. 2002; Zou & Gong 2003).

From 1999 to 2000, an outbreak of velogenic viscerotropic NDV occurred in a single commercial flock as well as village chickens in a relatively small geographic area of the KwaZulu-Natal province. The pathogen was identified as a genotype VIId virus. For almost 3 years afterwards there were no reports of ND outbreaks, nor were any virulent viruses isolated by national laboratories. Then, towards the end of September 2003, a velogenic ND virus was isolated from village chickens near Pietermaritzburg. This was close to the locations of the outbreaks of 1999 and 2000, but the infection did not seem to spread. The 2003 genotype VIId strain was phylogenetically linked to the 1999/2000 isolates (Abolnik et al. 2004).

Almost exactly 1 year later, in October 2004, an outbreak of genotype VIId started in commercial flocks of the Camperdown/Richmond districts. Although the index case was a commercial farm, workers reported that a disease with similar symptoms had been killing fowls in their villages for several weeks prior. A sample was obtained for testing. It was shown to be a genotype VIId strain, almost identical to the isolate from the commercial farm on which the village workers were employed. The spill-over was attributed to poor on-farm biosecurity practices. Interestingly, around this time a separate sub-genotype VIId was introduced into the outbreak region in KwaZulu-Natal (KZN); four isolates shared a very recent common ancestor with two Chinese goose viruses isolated in 2001. This genotype VIId subgroup circulated concurrently with the epidemic genotype VIId strain that had arisen in 1999/2000 but appeared to be displaced within months and did not spread beyond the KZN province.

After December 2004, genotype VIId spread northwards along the major poultry-producing corridor between KZN and Gauteng/North West provinces, affecting both commercial and backyard flocks. Outbreaks were usually associated with poor vaccination practices although sometimes mortalities were reported in well-vaccinated flocks. The index case in the northern region appears to have been contaminated hatching eggs sourced from a supplier in KwaZulu-Natal. Over the next few months, the disease spread westwards to the Northern Cape, southwards to the Cape provinces and eastwards into Mpumalanga province. The epidemic peaked in September 2005, and by 2006, it had begun to subside in most regions, except for the Western and Eastern Capes where cases were still increasing. The Western and Eastern Cape outbreaks were characterised by at least three separate introduction events from northern regions as established by mapping specific genetic markers. During the epidemic in commercial and backyard chickens, mortalities were recorded in peafowl, hadeda ibis chicks, geese, ostriches, pheasants and doves (Abolnik 2007). No cases of genotype VIId were detected in South Africa after 2012 (unpublished laboratory data).

### Genotype VIIh (2013 to present)

Genotype VIIh has caused outbreaks in the poultry of Bali, Indonesia and Malaysia since 2007, and an isolate was also recovered from a wild egret in China (Dimitrov et al. 2016). Genotype VIIh has since reached southern Africa. In December 2011 it caused an outbreak in vaccinated broilers in Mozambique, where it still persists (Mapaco et al. 2016). In late August 2013, genotype VIIh was diagnosed in a flock of commercial layers in Lephalale, Limpopo province and in a flock of free-range broilers near Potchefstroom, North West province. By October 2013, the strain had spread to the Free State province and had been confirmed in Zimbabwe. By November 2013, the outbreaks had spread to Gauteng and reached KwaZulu-Natal by July 2014 and Mpumalanga in October 2014. The South African genotype VIIh viruses share a very recent common ancestor with strain Sukorejo/019/10, with 98% nucleotide sequence identities across the complete genome (data will be published elsewhere). The Sukorejo virus was isolated during an outbreak in commercial poultry that circulated in Indonesia in 2009 and 2010 (Xiao et al. 2012). Imported poultry, poultry products or exotic birds from South East Asia are the likely source of genotype VIIh in the southern African region and while it is likely that genotype VIIh spread to South Africa across a neighbouring international border, the exact epidemiological source remains unclear.

## Conclusion

Small-scale poultry production has enormous potential to stimulate the socio-economic growth of resource-poor households. Chickens provide a good source of protein and ready cash for households, help to sustain the rural economy and contribute to the prevention of urban migration, yet ND is the single largest threat to poultry production in Africa. The commercial sector also bears a huge economic burden as a result of the disease. In addition to production losses incurred during outbreaks and the chronic costs of control through vaccination, with a constant ND-endemic status valuable trade and export markets remain inaccessible.

**FIGURE 1:**
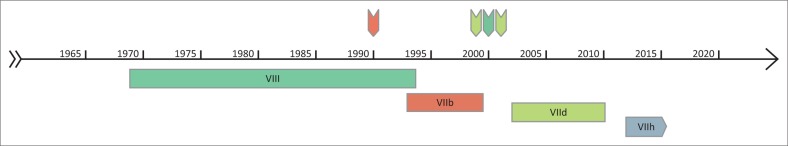
Timeline of Newcastle disease virus outbreaks in South Africa since the late 1960s, which were caused by successive outbreaks of genotypes VIII, VIIb, VIId and VIIh, respectively.

From genotype VIII in the 1960s and 1970s, to genotype VIIb in the 1990s, genotype VIId in the 2000s and now genotype VIIh, each genotype displaced its predecessor and circulated for approximately a decade (Figure 1). This is an unusual trend, since in other ND-endemic regions (e.g. East Asia) numerous genotypes are in co-circulation at any time. Each of South Africa’s outbreaks was caused by exotic strains of ND introduced at periodic intervals. The illegal importation of poultry, poultry products or exotic birds was the likely source of infection. Genotypes VIIb and VIIh index cases were found inland, but genotype VIId was likely introduced through the port of Durban, by illegal harbour swill dumping (Abolnik 2007). Infected swill has indeed been implicated in the introduction of infectious diseases into South Africa before. In 2000, foot and mouth disease was introduced into KwaZulu-Natal by contaminated swill (Brückner et al. 2002). Classical swine fever was probably also introduced from Asia into the Western Cape in 2005 by illegal swill feeding of pigs (Penrith 2013). Any presence of the disease in neighbouring counties is likely to spread throughout the region, as people, vehicles and poultry move across the borders. Once ND is established in the South African commercial sector, it spreads rapidly along major transportation routes via movement of infected poultry and/or fomites. This is facilitated firstly by the large commercial producers themselves who transport hatching eggs and day-old chicks between their operations, buy in supplemental eggs at pack stations on commercial layer farms, frequently transport manure between sites in uncovered trucks, and secondly by the cull buyers, that is vendors of spent layer hens.

In developed countries, commercial chickens have almost no resale value after their usefulness has expired. In South Africa, however a spent layer hen with a replacement value of about R50 (±$4.40) can be sold for about R35 (±$3.10) allowing the farmer to recoup most of the expense. Cull buyers pick up batches of thousands of birds with trucks and transport them to central depots. Some large commercial operations even have their own cull depots and in this way birds from different farms are brought together. From the depot, fowls (perhaps several hundred or less) are sold to smaller buyers, who then supply birds to informal markets including townships and rural areas. Where the market is good, large cull buyers will often travel over long distances, for example, between Gauteng and the Western Cape or KwaZulu-Natal. Therefore culls that originate in the Cape can very easily, and are commonly, be sold in KwaZulu-Natal. Interestingly, cull chickens are not significantly cheaper than frozen meat, but these older chickens are coveted because consumers consider them to be tastier. Therefore, in South Africa, a layer hen still has a value at the end of her production life. This makes the layer market very competitive as about 25 million layer hens are in production in South Africa at any given time (Southern African Poultry Association 2012). The cull-buyer industry is legal, albeit largely unregulated and uncontrolled. A positive consequence of a cull-buyer system is that it appears to have limited the need for large live bird markets in South Africa. Live bird markets are key epidemiologic points for the spread of ND and other dangerous diseases like highly pathogenic avian influenza in other developing countries in Africa and Asia.

Commercial live vaccines such as LaSota, VG/GA, Clone 30 and B1 (Genotype II) are applied for prevention and control of ND in South Africa, yet outbreaks continue to occur. These classical vaccines have been experimentally demonstrated *in vivo* to provide complete protection against clinical disease and mortality induced with virulent strains (including genotype VII) that are antigenically mismatched to the vaccine strain, and still either significantly reduce or completely prevent shedding. Even though some of these experiments were conducted with ‘sub-optimal’ vaccine doses (Dortmans, Peeters & Koch 2012), titres of 10^4^ EID_50_/mL live vaccine applied under ideal laboratory conditions still equates to a significant number of live replicating viruses. Current mass-immunisation methods involving vaccine application by spraying or drinking water are possibly insufficient to guarantee that each bird in the house receives an adequate vaccine dose (Dortmans et al. 2012). Concurrent infection with immunosuppressive pathogens may also contribute to poor immunity levels so facilitating ND viral spread (Jeon et al. 2008). ND may be spread in supposedly well-vaccinated flocks and maintained over a period of years (Dortmans et al. 2012). The efficacy of transmission from birds shedding low ND virus titres under field conditions remains to be investigated. Clearly what is urgently needed in South Africa is stricter implementation of good biosecurity measures as well as enforcement of the existing control policies to prevent newly-introduced exotic strains from becoming epidemics. The ultimate goal should be to attain ‘ND-free’ status.
